# Selective Visual Attention in ADHD: A Narrative Review

**DOI:** 10.1007/s11910-025-01435-5

**Published:** 2025-07-24

**Authors:** Jennifer L. Klein, Harriet A. Allen, John Clibbens, Amy Cook, Virginia Amanatidou, Eirini Mavritsaki

**Affiliations:** 1 Department of Psychology, Birmingham City University, Curzon Building, 4 Cardigan Street, Birmingham, B4 7BD UK; 2 Department of Psychology, University of Nottingham, University Park, Nottingham, NG7 2RD UK; 3 Herefordshire and Worcestershire Health and Care National Health Service Trust, 2 Kings Court, Charles Hastings Way, Worcester, WR5 1JR UK

**Keywords:** ADHD, Selective attention, Bottom-up, Top-down, Visual search

## Abstract

**Purpose of Review:**

Attention-deficit/hyperactivity disorder is a common neurodevelopmental disorder characterized by impairing levels of inattention, hyperactivity and impulsivity that adversely impact functioning across social, academic/occupational and home settings. While the name of the disorder implies obvious difficulties in attention, research has struggled to consistently identify a precise neurocognitive marker. This article aims to characterize the functioning of selective visual attention in ADHD by reviewing previous studies that compare individuals with ADHD and healthy controls at the behavioral and neural levels using single-frame visual search tasks.

**Recent Findings:**

Past research indicates both bottom-up (stimulus-driven) and top-down (goal-driven) attention across both time and space are likely affected in ADHD. However, more research is needed to illuminate the specific mechanisms involved.

**Summary:**

Ultimately, this narrative review aims to highlight the importance of studying selective visual attention in ADHD to explain the heterogeneous symptoms and impairments of this complex disorder, as well as to build a stronger bridge between the high-level behaviors of ADHD and their underlying neurobiological mechanisms.

## Introduction

Attention-deficit/hyperactivity disorder (ADHD) is a common mental disorder characterized by developmentally inappropriate and impairing levels of inattention, hyperactivity and impulsivity [1]. ADHD affects approximately 8.0% of children and adolescents [2] and between 3.1 and 6.7% of adults [3–5], making it one of the most prevalent mental health disorders worldwide [6–8]. ADHD has been traditionally characterized as a neurodevelopmental disorder that begins in childhood and resolves by adulthood [9, 10]. However, the symptoms and impairments of ADHD continue to persist into adulthood for somewhere between 65% and 90% of cases [11, 12]. Furthermore, recent evidence supports the existence of late-onset ADHD, where symptoms first arise after the age of 12 [13, 14], thus challenging the characterization of ADHD as a neurodevelopmental disorder [10].

ADHD is considered to be a highly complex and heterogeneous disorder [15, 16], as affected individuals vary substantially in their genetic risk [17], environmental contribution [18], and profile of neurocognitive impairments [52]. Individuals with ADHD demonstrate differences in working memory [19] response inhibition [46], and decision-making [20], among other cognitive functions [52]. Ultimately, those with ADHD are at a higher risk for functional impairments and adverse outcomes, including lower educational and occupational attainment [21, 22], problems with peer and romantic relationships [23, 24], and lower overall quality of life [25, 26]. Furthermore, children and adults with ADHD are more likely to have comorbid mental and somatic (e.g., obesity) problems [27–29], and are at an increased risk for self-harm/suicidal ideation [30, 31] and criminality [32]. Accordingly, there exists a distinct need to develop a thorough understanding of what deficits occur ADHD, how they arise, and how this translates to impaired functioning in everyday life.

### Understanding the “Deficit” in Attention-Deficit/Hyperactivity Disorder

One of the early theories of ADHD proposed that symptoms associated with the disorder arise from a core deficit in arousal [33]. Specifically, Satterfield et al. [33] found that children with ADHD who had low levels of central nervous system (CNS) arousal had the highest levels of disruptive behaviour in the classroom and were the best responders to stimulant medication. The authors went on to reference work that stimulant-treated ADHD children show improved attention and performance on psychological tests - referring to Conners’ Continuous Performance Task (CPT; [34, 35]). Indeed, one of the more commonly used neurocognitive measures to assess for deficits in ADHD is the CPT [36, 37]. There are several variants of the CPT, but for all, the goal is to consistently identify a particular target stimulus among distractors (where items are presented successively) over a prolonged period of time - usually 15 minutes or more. Indeed, compared to non-ADHD controls, children and adults with ADHD typically perform worse on the CPT with longer, more variable RTs and higher error rates [38, 39]. However, this is not always the case, as some studies have reported no significant differences between ADHD and non-ADHD individuals [40]. Furthermore, while recent research also provides support for the idea that the CPT is able to effectively identify symptoms of inattention [37], there are still mixed findings as to the CPT’s sensitivity (i.e., ability to correctly identify ADHD individuals) and its overall diagnostic utility [37, 41, 42].

Other ADHD research has focused on response inhibition as the core deficit of the disorder. Barkley [51] proposed that the wide variety of impairments associated with ADHD arise from a core deficit in behavioral inhibition, or the ability to stop an initiated behavior in furtherance of a specific behavioural goal [43, 44]. This deficit in response inhibition is often theoretically tied to hyperactive/impulsive symptoms (rather than inattentive symptoms), and in CPT and Go/No-Go tasks, deficits are thought to be reflected behaviorally by increased commission errors (false alarms). In another popular measure, the Stop Signal Reaction Time (SSRT) task, response inhibition deficits are reflected in longer RTs [43, 45]. Indeed, children, adolescents and adults with ADHD all perform worse on measures of response inhibition [46–50]. Barkley [51] proposed that this deficit in behavioral inhibition gives rise to the wide variety of deficits in executive functioning. However, empirical evidence supporting this specific relationship is lacking. In a review of 34 meta-analyses that compared performance between ADHD and non-ADHD individuals on a wide variety of neurocognitive domains (e.g., set shifting, WM, RT variability, etc.), Pievsky and McGrath [52] found that the size of response inhibition deficits were moderate (0.52) and, rather than being the deficit with the largest effect (as would expect if it is indeed the core deficit), was nearly identical in size to other deficits [52].

The notion that ADHD is characterized by deficits in arousal and/or response inhibition seems intuitive. Indeed, the proposals put forth by Satterfield et al. [33] and Barkley [51] highlight a key challenge present in ADHD research: the vocabulary surrounding impairments appears to diverge, as the terms “arousal,” “vigilance” and “sustained attention,” are typically associated with the inattentive subtype, while “response inhibition” is associated with the hyperactive/impulsive subtype. For example, much of the research in ADHD expresses a difficulty in the ability to reconcile two symptoms dimensions that are viewed as paradoxical [10, 53–56], with the inattentive subtype reflected by a lack of responding (omission errors) and the hyperactive/impulsive subtype indicated by an inappropriate level of over-responding (commission errors; [37, 57]). Naturally, when individuals with ADHD demonstrate difficulty in sustained attention or vigilance tasks, this is often interpreted within frameworks that support the idea of a core deficit of low arousal/activation (e.g., Cognitive Energetic Model; [58, 59]. Similarly, in tasks of response inhibition, deficits are often interpreted in terms of Barkley’s [51] behavioral inhibition model [52, 60].

However, it is important to return to the original text and review the authors’ definitions of these terms and the context in which they were offered. For example, at the time of Satterfield’s [33] low arousal theory, children with ADHD were diagnosed as having “hyperactive child syndrome.” Indeed, the participants in Satterfield et al.’s [33] study were characterized as having, “[A] chronic symptom pattern of hyperactivity, distractibility, excitability and impulsivity” (Satterfield et al. [33], p. 839). Thus, it is likely that, today, these children would be characterized as the hyperactive/impulsive subtype [1]. Importantly, in explaining the relationship between low levels of (CNS) arousal (as measured by skin conductance levels) and high levels of disruptive classroom behaviour in these children, Satterfield et al. [33] offered the following explanation: “Lack of inhibitory control over sensory function could be expected to result in easy distractibility, with the low aroused child responding to irrelevant stimuli as ready as to relevant stimuli” (Satterfield et al. [33], p. 842). This relationship between low arousal and hyperactive/impulsive behavior blurs the seemingly clear dichotomy between subtypes, impairments, and causal mechanisms – how could low arousal cause both deficits in under-responding (inattentive) *and* over-responding (hyperactive impulsive)?

### Selective Visual Attention in ADHD

Some of the apparently contradictory nature of ADHD may be lessened by understanding deficits in terms of selective attention and by focusing on the nature of competitive interactions at the neural level. *Selective attention* refers to the ability to selectively process relevant information while simultaneously ignoring information that is irrelevant and potentially distracting [61, 62]. Importantly, this definition of selective attention describes the behavioral result that arises from biased competition neural computations which occur within and across the cortical hierarchy [61, 63, 64]. This selection occurs across the cortical hierarchy at multiple levels of abstraction – so whether it’s low-level sensory information, or higher-order working memory representations or rule structure representations, competition occurs at every level of the cortical hierarchy [64, 65]. Thus, when there is a shared focus on the competitive interactions that occur at the neural level, it is easier to conceptualize how inefficient behaviour might arise from selection of the wrong stimuli, rule, or motor response.

Furthermore, by understanding deficits in ADHD through selective attention, a more cohesive view of both inattentive and hyperactive/impulsive symptoms and impairments can be achieved. The prefrontal cortex (PFC) is a critical site in cognitive control and the regulation of attention [66, 67]. Within the PFC, two key catecholamines - dopamine (DA) and norepinephrine (NE) - contribute to maintaining the region’s extremely sensitive neurochemical environment [68]. Specifically, these catecholamines are crucial to maintaining an optimal levels of arousal, which in turn support an optimal signal-to-noise ratio (SNR; [69]. Optimal SNR supports the ability to maintain complex representations online in working memory, and these representations serve as the source of top-down attention biases [70]. Importantly, optimal SNR occurs at moderate levels of arousal. At low levels of arousal (e.g., during drowsiness or boredom), ADHD-like symptoms appear, such as impaired working memory, increased distractibility, poor impulse control and motor hyperactivity [71]. Similar symptoms - in particular, impaired working memory - also appear at abnormally high levels of arousal (e.g., during stress) as well [68, 72]. Several of the genes associated with ADHD involve catecholamine neurotransmission, including NE and DA receptors and transporters [73–76], and dopamine beta-hydroxylase (DBH), the enzyme required for NE synthesis [77, 78]. Furthermore, the gold-standard treatment for ADHD is psychostimulant medication (e.g., Ritalin, Adderall; [79]). Non-stimulant medications are also available but are considered less efficacious [80, 81]. Both stimulant and non-stimulant medications for ADHD act by raising the amount of catecholamine neurotransmission in the PFC [82, 83]. Thus, the seemingly paradoxical symptoms could be explained by this “inverted U,” relationship in the PFC, where deficits in ADHD may arise not from a definitive lack of attention, but a difficulty in regulating levels of arousal, where too little or too much arousal reduces the ability to control interference and thus impairs competitive interactions.

**Fig. 1:**
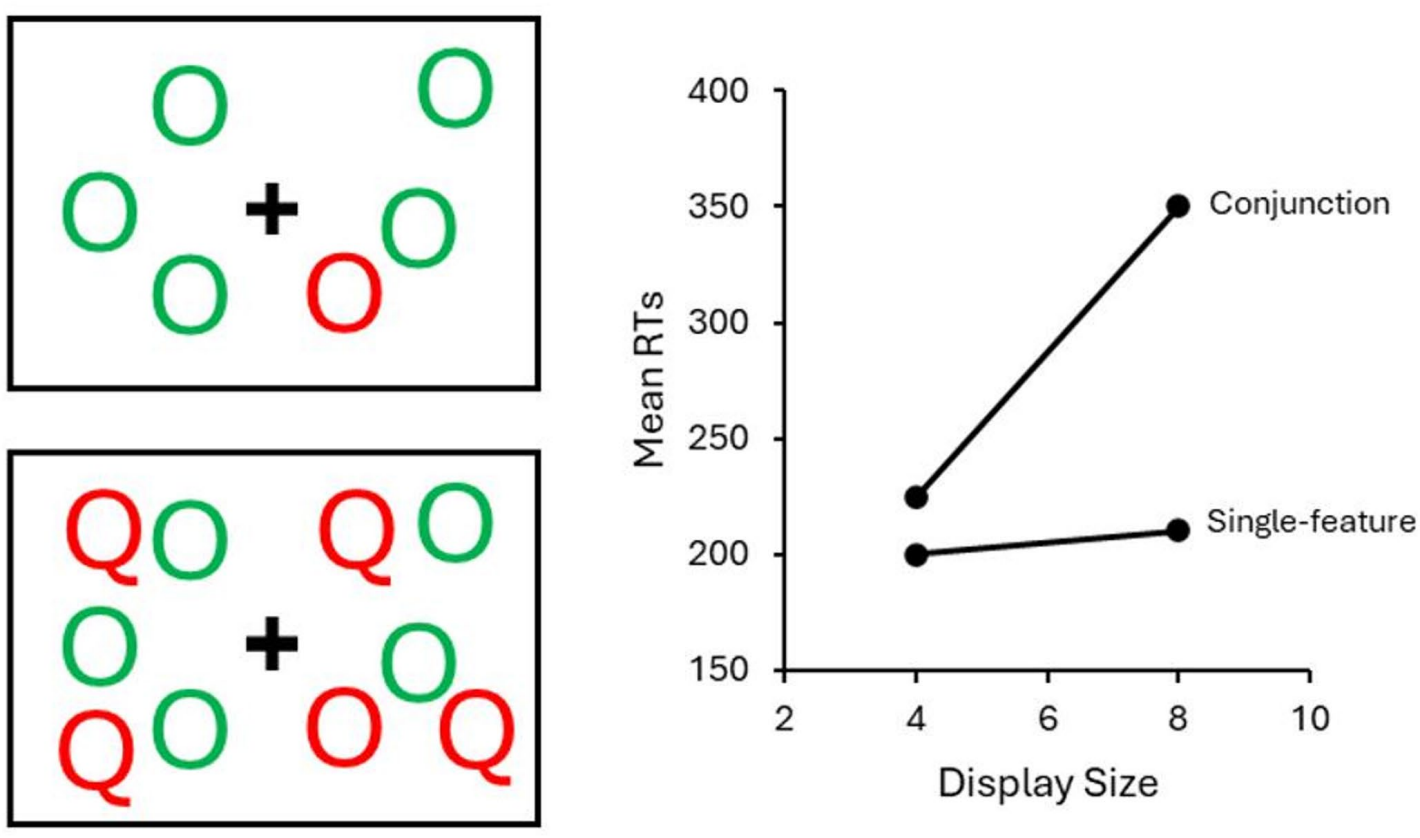
Single-feature and Conjunction Search Displays and Slopes. In the single-feature search (top left), the target letter “O” appears to pop out from the green letter “O” distractors in the display. In the conjunction condition (bottom left), the target red letter “O” is more difficult to find among the green letter “O”s and red letter “Q” distractors. On the right are the typical flat and steep slopes of the RT function for these search conditions (respectively)

In the visual domain, selective attention has famously been likened to a spotlight that enables enhanced processing of stimuli that falls within the location of its “beam” [84]. This idea was subsequently expanded by Feature Integration Theory (FIT; [85]), which proposed attention binds separable visual features (i.e., color, shape) into whole objects. FIT proposes that, during an initial “pre-attentive” stage, perceptual information is processed in parallel and basic visual features are automatically encoded in different parts of the visual cortex. In a subsequent “attentive” stage, the serial application of selective attention facilitates the binding of these visual features in a “master map,” thus allowing the selected features to be identified as a perceptual object at higher levels of processing. Support for FIT was provided primarily by visual search tasks (see Fig. 1), which require subjects to search a display of randomly positioned “distractor” items to identify a pre-determined target [86, 87]. The number of distractors surrounding a target in a display, referred to as the display size (or set size) varies from trial to trial. Performance in visual search tasks is measured by the mean reaction time (RT) as a function of the display size, i.e., the slope of the RT function [88, 89]. Two types of visual search conditions, known as single-feature and conjunction, were used as evidence of pre-attentive and attentive stages, respectively [85]. In single-feature search, the target item appears to “pop out” from the search display, as it is defined by a difference of one feature from its distractors (e.g., a red letter “O” target among green letter “O” distractors). In a conjunction search, the target is a conjunction of two features, and distractors belong to one of two groups that are defined by one of the target features (e.g., a red letter “O” target among green letter “O” and red letter “Q” distractors). According to FIT, the stage of processing at which search operates is reflected in the search slope. In a single-feature search, slopes are typically quite flat, as RTs are not affected by the number of distractors present. This reflects the pre-attentive stage, where there is an absence of any focused attention. In conjunction search, slopes are steep, as RTs increase linearly with the number of distractors, implying the application of effortful attention [90–92].

Much of what was proposed by FIT has since been revised [86–95]. Wolfe and colleagues [96, 97] proposed in their Guided Search Model (GSM), that, rather than random allocation, visual attention is guided across space to the most likely location of a target item. This guidance is based on an observer’s prior knowledge of the target item’s features (top-down signals), its inherent saliency and its similarity to other items (bottom-up signals; [89, 92]). Additionally, while FIT and GSM propose the distinct separation between “pre-attentive” and “attentive” stages, research has demonstrated that the effects of attention can be seen in the early visual cortex, suggesting that visual perception and attention cannot be parsed apart so easily [98]. Despite this, FIT and GSM provide an important framework through which we can more easily distinguish between bottom-up attention - the process through which attention is captured through an object’s inherent saliency and top-down attention - the process by which attention is effortfully allocated based on an internally-held goal [92]. Finally, top-down and bottom-up attention has also been studied extensively using single-cell recordings in macaques during visual search tasks [98–103], thus providing an important bridge between the neural and behavioral levels that could be key in identifying the biological mechanisms of high-level behavior.

#### Bottom-Up Attention in ADHD

Much of the previous work investigating deficits in ADHD has focused on performance in effortful, top-down control. However, bottom-up attention may also be implicated in this disorder. Indeed, in single-feature (i.e., pop-out) search tasks, individuals with ADHD are often significantly slower [104–110], more variable in their responses [104, 107–111], and/or less accurate [104–106, 108, 112] than their non-ADHD peers. However, these findings are not always consistent for either RTs [112–114] or for accuracy [107, 109–111, 113–115]. While reaction time (RT) and accuracy are important measures, most studies using pop-out search fail to examine the RT-display size function (i.e., search slope), which is critical to understanding the mechanisms of search [89]. However, the few studies that did examine search slopes [105, 108, 113, 115] found that ADHD and non-ADHD slopes did not significantly differ, suggesting an intact attention to a visually salient target stimulus. Ultimately, while the limited evidence on search slopes indicates that the fundamental mechanisms of bottom-up attention may be operational in ADHD, their slower, more variable, and less accurate performance is indicative of underlying difficulties in attentional processing.

Several studies have also demonstrated atypical neural signatures of bottom-up attention in ADHD. For instance, it has been shown that children with ADHD also demonstrate higher amplitudes in the P1 ERP component during pop-out search compared to their non-ADHD peers [107]. The P1, a positive-going waveform in the event-related potential (ERP) that occurs about 100 msec after stimulus onset, is thought to reflect the early perceptual processes that occur during the initial feedforward sweep of the visual field and is modulated by top-down attention [116]. The authors proposed that increased P1 amplitudes in ADHD during pop-out search may reflect an overreliance on bottom-up attention as a result of less control over top-down attention [107]. Furthermore, during pop-out search, children with ADHD have reduced amplitudes of the N2pc component, a negative-going waveform in ERP that occurs at about 200–300 msec after stimulus onset [106, 109, 110]. As the N2pc is thought to index attentional selection of a target item [116], these findings indicate that this process is impaired even when that target is highly salient. In an effort to better understand how pop-out targets are processed in ADHD, Li et al. [106] applied an ERP-based multivariate pattern decoding approach to ERPs collected during a pop-out search. They found that, at around 200 msec after stimulus onset, children with ADHD were less precise in their ability to represent the location of target item compared to their non-ADHD peers. Furthermore, children with ADHD were significantly slower to detect the target item during search. Interestingly, there was also a significant correction found between N2pc amplitudes and target decoding accuracy in non-ADHD children, but this correlation was not present in ADHD children, suggesting that, in ADHD children, the encoding of the target location is achieved without the N2pc component [106]. Finally, Cross-Villasana et al. [117] found that adults with ADHD showed delayed N2pc peak onsets and peak latencies compared to non-ADHD adults; however, there was no difference in N2pc amplitudes in adults, suggesting that bottom-up attention difficulties in ADHD may change over the course of development [117].

Functional magnetic resonance imaging (fMRI) in children with ADHD during a pop-out search showed that, compared to non-ADHD peers, there is greater activation in the fronto-parietal regions (bilateral temporoparietal junction [TPJ], right inferior frontal gyrus [IFg] and middle temporal gyrus [MTg]), suggesting that pop-out search in ADHD is less efficient and requires more effort that recruits top-down involvement [111]. Taken together, these findings indicate the mechanisms used to locate and identify salient target items may indeed differ in ADHD. However, these unique mechanisms may ultimately yield behavioral performances that can often be indistinguishable from their non-ADHD peers.

#### Top-Down Attention in ADHD

##### Across Space

In order to achieve behavioral goals, the effortful control of attention (i.e., top-down attention) must be applied in a serial manner to items in a cluttered scene, where irrelevant items are successfully ignored and prior knowledge of the target’s features act as guidance [118, 119]. One method used to explore the process of ignoring irrelevant information – particularly when it is salient – is to include a singleton distractor in a standard single-feature search. Here, a single distractor within the display contains a feature that is not shared by any other item. For instance, in a search display where the target is a white circle surrounded by white square distractors, a green square singleton distractor captures attention strongly, and thus top-down control is required to ignore this salient but irrelevant item after initial capture [120–123]. Several studies that have implemented this method to compare distractor suppression in ADHD have found that children with ADHD are significantly slower [109], more variable [109] and less accurate [109, 112] than their non-ADHD peers. In a study by Wang et al. [109], the distractor positivity, or P_D_, was recorded in children with and without ADHD during a singleton distractor search task. The P_D_, which is elicited over the posterior brain regions contralateral to the side where singleton distractors appear, is thought to reflect the active suppression of these distractor items [124, 125]. Wang et al. [109] found that children with ADHD had smaller P_D_ amplitudes compared to their non-ADHD peers, indicative of weaker top-down suppression of irrelevant distractors.

Further evidence of atypical top-down suppression in ADHD has been shown by Zhu et al. [112], who examined the activation and functional connectivity of the inferior parietal lobule (IPL) in children with and without ADHD during a standard single-feature and a singleton distractor task. At the behavioural level, children with ADHD demonstrated worse accuracy for both tasks. At the neural level, fMRI data showed reduced activation in the IPL for both tasks as well. Importantly, during the singleton distractor task, connectivity analysis showed that children with ADHD demonstrated stronger functional connectivity of the right IPL and IFg. The reduced IPL activation but stronger functional connectivity with frontal regions in the singleton distractor task may indicate a unique strategy in ADHD where frontal regions are recruited to compensate for attentional difficulties [112].

Other studies have used traditional conjunction search to probe top-down attention differences in ADHD. Again, children with ADHD have been shown to often be significantly slower [105, 108, 115, 126, 127], more variable [108], and less accurate [105, 108, 126, 128] than their non-ADHD peers. However, findings are not always consistent for either RT [111, 113, 114] or accuracy [111, 113–115]. One study has even shown that children with ADHD were significantly faster than their non-ADHD peers in conjunction search [129]. Similar to single-feature search, the RT-display size function (search slope) should also be analyzed in conjunction search to examine the mechanisms of search [89]. Search slope has been examined by a few studies [105, 108, 113, 115, 126–128]. Of these, three have found that slopes were significantly steeper in children with ADHD [105, 127, 128], suggesting a potential impairment in the ability to allocate effortful attention across space in a serial manner. Interestingly, a literature review by Mullane and Klein [130] compared visual search performance between children with and without ADHD found that, although groups performed similarly under single-feature search conditions, children with ADHD were less efficient in conjunction search tasks, particularly in overly easy and overly complex search displays. These findings suggest that children with ADHD have difficulty allocating effortful attention under both “boring” and stressful conditions, but difficulties disappear at optimal levels of stimulation [72, 130].

A few studies have also provided some insight into differences that occur at the neural level in effortful search tasks in ADHD. For instance, Taylor et al. [114] examined the P300, a positive-going waveform in the ERP that occurs 300–500 ms after stimulus onset thought to index attentional engagement [131], during a conjunction search. The authors found that, compared to non-ADHD children, children with ADHD had significantly shorter P300 latencies, suggesting that effortful, serial processing is less controlled in ADHD. Furthermore, they found that the shorter P300 latencies in ADHD continued to arise even when medicated with both low- and high-dose psychostimulants [114]. Using fMRI, Booth et al. [126] found that children with ADHD had reduced activation during a conjunction search in several regions, including the right superior parietal lobule (SPL), right cuneus, right MTg and left fusiform gyrus. Finally, O’Conaill et al. [111] also found that children and adolescents with ADHD also displayed hypoactivation within the temporal lobe compared to their non-ADHD peers during a conjunction search task. However, children with ADHD also demonstrated increased activation in the TPJ during this search. While the existing behavioral and neural evidence is limited, there is indeed an indication that differences in effortful, serial attention occur in childhood ADHD. However, more research is needed to characterize these differences in children with ADHD, and to determine if these continue on in adulthood.

##### Over Time

While single-feature (with or without singleton distractors) and conjunction search are able to aid in our understanding of ADHD-related differences in how attention is allocated across space, attention must also operate effectively within the temporal realm to produce efficient behavior. A variation of conjunction search called *preview search* has been widely used to explore time-based attention. In a typical preview search condition, the final display is identical to that of a standard conjunction search (e.g., a red letter “O” target among green letter “O” and red letter “Q” distractors). However, unlike a conjunction search, one set of distractor items (e.g., the group of green letter “O”s) is displayed for a short time (a minimum of about 450 msec; [132, 133] before the second set of distractor items and target item appear alongside. Although the final search display of a preview search is identical to that of a standard conjunction search, the “previewing” of half the distractors produces significantly more efficient search that can even be as efficient as if only new items were presented (e.g., a single-feature search; for reviews, see [134] and [135]). Initially, it was argued that the preview benefit arises as the result of a top-down attentional mechanism, referred to as visual marking, whereby old items are suppressed to allow new items to be prioritized [134–136]. More recent research has highlighted the contribution of a more bottom-up attention mechanism, temporal binding (the binding of features by common onset), using preview gap search [137, 138].

Time-based attention differences in ADHD has been previously investigated through a series of studies by Mason et al. [108, 113, 139]. Using preview search, Mason et al. [108] found that children with ADHD did not differ significantly from their non-ADHD peers. Both groups were able to generate preview benefits, with search more efficient than the typical conjunction task (although not as efficient as single-feature; [108]). In a follow-up study, Mason et al. [113] found again that both groups were able to generate a robust preview benefit (here, as efficient as single-feature).

In their follow-up study, Mason et al. [113] also used a preview search condition that incorporated a singleton in the final display to examine how the suppressive effects of visual marking carry over from preview to final displays. In one condition, a preview display consisting of green vertical rockets was displayed before the remaining items - red horizontal rocket distractors, one singleton green vertical rocket, and the red vertical rocket target appeared in the final display. A previous study using this singleton distractor preview condition found that adults experience less interference (i.e., faster RTs) in this condition due to the “carry-over” inhibition of preview-item features [140]. Surprisingly, both ADHD and non-ADHD children experienced *more* interference (i.e., slower RTs) in this condition. The authors proposed that children may find the top-down control needed to successfully mark old items demanding, thus leading to a loss of the effortful inhibition in the final display. In a second condition, the same preview display was followed by red horizontal rocket distractors and a green vertical rocket target. Similar to adults, children’s performance was slower in this singleton target condition [140]. However, children with ADHD were significantly slower in this condition compared to their non-ADHD peers. The authors proposed children with ADHD struggle to effectively manage their top-down control across time in order to switch from a negative set (e.g., inhibition of old items) to a positive one (e.g., selection of the target; [113]).

In a third and final follow-up study by Mason et al. [139], the authors used a rapid serial visual presentation (RSVP) task to further understand how children with ADHD process distractors over time. They found that both children with ADHD and their non-ADHD peers experienced attentional capture by a singleton distractor that shared its color (red) with the target item. However, unlike their non-ADHD peers, children with ADHD were distracted by a singleton that did not share its color with the target item. The authors proposed that these results also demonstrated that children with ADHD experience difficulty in the ability to maintain top-down control, particularly when required to do so over time [139]. Thus, in addition to previous literature that indicates children with ADHD struggle to allocate attention effectively across space, this difficulty also extends to the temporal realm. Furthermore, these studies demonstrate that while children with ADHD are able to generate a preview benefit, they are likely impaired in some of the key functions that allow for efficient visual marking. As such, further research is needed to understand how time-based attention operates in ADHD.

## Conclusion

While earlier research in ADHD did point to deficits in selective attention, particularly in the visual domain [33], terminological differences (e.g., referring to it as “arousal”) may cause this research to often be overlooked. Furthermore, other research in ADHD has pointed to various core deficits in sometimes conflicting neurocognitive deficits of both over- and under-responding (i.e., omission vs. commission errors; [45, 53]). In the present review, we suggest that a more comprehensive understanding of deficits in ADHD can be reached by viewing them through the lens of selective attention. The importance of selective attention in ADHD is primarily supported by evidence at the neural level, where reduced neurotransmission of NE and DA, particularly within the PFC, which are critical to the proper functioning of arousal and SNR, both of which support the complex mechanisms that enable us to efficiently allocate our attention across both time and space, ultimately producing efficient, self-regulated behavior [68, 72, 141]. A large body of research has been dedicated to understanding the mechanisms of selective visual attention at the behavioral, network and neural levels [61, 62, 85, 142]. Visual search tasks have yielded significant insights into the mechanisms of attention [86, 87]. However, search tasks have been widely underutilized in ADHD research.

Indeed, visual search research in ADHD is limited and inconsistent. However, at present, the evidence shows individuals with ADHD demonstrate several deficits in selective visual attention. For one, children with ADHD often experience difficulties in more bottom-up (stimulus-driven) attention as measured by single-feature (i.e., pop-out) search. This is supported by neuroimaging methods that show smaller ERP amplitudes and hypoactivation in posterior regions that support attentional functioning [106, 109, 110, 112], and some increased activation in frontal areas typically associated with top-down control [111], possibly suggesting reliance on recruitment of other areas to compensate for weaker attentional function. These difficulties also appear to extend to top-down attention, specifically in the top-down suppression of salient-but-irrelevant distractors [102, 112], and this difficulty in top-down suppression extends not only in spatial domain, but likely the temporal domain as well [108, 113, 139].

Only a few studies have investigated the impact of stimulant medication on visual attention functions in ADHD, despite the potential for valuable insights using this methodology. As previously mentioned, Taylor et al. [114] surprisingly found that the shorter P3 latencies observed during ADHD children’s conjunction search performance did not change with administration of either low- or high-dose stimulants. More recently, Guo et al. [143] used a double-blind placebo controlled cross-over design to investigate the impact of first-dose methylphenidate on behavioral performance and neural indices in a single-feature search. They found that behavioral performance – measured in error rates and mean RTs – improved as a result of stimulant medication. Moreover, the administration of stimulants increased the amplitude of both the N2pc and P300, which are thought to index the processes of selection and top-down control of attention (respectively; [116; 131]). As stimulant medications act to raise levels of NE and DA in the brain (particularly in the PFC [82,83]), future research examining how stimulant medication affects visual search performance and its associated neural indices may yield insight into how differences in neurotransmission in ADHD translates first to measurable neurocognitive markers (like selective attention) and then ultimately to a wide range of behavioral symptoms [143]. 

Although, at present, the evidence of selective visual attention difficulties in ADHD is limited, there are still some important implications for the treatment of ADHD from studies of visual search performance. For instance, much of the focus in the ADHD research centers around deficits in more executive functioning and effortful control of attention [72]. Both behavioral and pharmaceutical treatments for ADHD are largely aimed at rectifying what is often considered to be a core deficit in top-down functioning [144, 145]. While effortful control is certainly a key area of interest in this disorder, findings from visual search tasks also point to difficulties in more bottom-up attentional processing. Accordingly, it is possible that some attentional problems in this disorder could actually arise from issues in perceptual processing, as the competitive interactions of attention can only occur when objects or locations are processed well enough to elicit competition in the first place [64]. Accordingly, resolving any issues at the perceptual level may be an important part of a larger treatment plan. Furthermore, rather than a core deficit in attention, some of the evidence from performance in visual search tasks suggest that individuals with ADHD struggle with the regulation of attention [130], Therefore, it may be beneficial to promote behavioral treatments that focus on developing self-regulatory skills and strategies.

Future research should focus on better characterizing selective visual attention deficits in ADHD, not only at the behavioral level using visual search tasks, but also through complementary methods such as neuroimaging, machine-learning, or computational modelling to better understand these deficits. For example, as it is shown here, neuroimaging during visual search tasks has shown that behavioral performance may not always be able to reveal differences that occur covertly at the neural level. Perhaps the use of compensatory strategies through recruitment of additional neural regions in ADHD may serve as a source of the inconsistency in behavioral results. Finally, future research should aim to characterize how selective visual attention deficits in ADHD may evolve and change over the course of development and into adulthood. Ultimately, there is significant potential in studying selective visual attention in ADHD, particularly when it comes to understanding how deficits at the neural level produce heterogeneous symptoms and impairments at the behavioral level. As such, more research is needed in this area.

## Key References

- Li D, Luo X, Guo J, Kong Y, Hu Y, Chen Y, et al. Information-based multivariate decoding reveals imprecise neural encoding in children with attention deficit hyperactivity disorder during visual selective attention. Hum Brain Mapping. 2023;44(3):937–47. 10.1002/hbm.26115.
-
  - By applying machine learning to ERP data, this article found that reduced the N2pc amplitude in children with ADHD may be related to the inefficient encoding of target items.
- Zhu Y, Luo X, Guo X, Chen Y, Zheng S, Dang C, et al. Functional reorganization of brain activity in children with attention-deficit/hyperactivity disorder: Evidence from the modulatory effect of cognitive demand during visuospatial attention task. J Psychiatr Res. 2023;166:17–24. 10.1016/j.jpsychires.2023.08.008.
-
  - This article found that worse performance in top-down distractor suppression is accompanied by reduced activation in the inferior parietal lobule but increased functional connectivity between this area and frontal regions, perhaps reflecting a compensatory mechanism that requires more top-down involvement.

## Data Availability

No datasets were generated or analyzed during the current study.
